# Stereotactic Radiation and Dual Human Epidermal Growth Factor Receptor 2 Blockade with Trastuzumab and Pertuzumab in the Treatment of Breast Cancer Brain Metastases: A Single Institution Series

**DOI:** 10.3390/cancers14020303

**Published:** 2022-01-08

**Authors:** Edy Ippolito, Sonia Silipigni, Paolo Matteucci, Carlo Greco, Francesco Pantano, Giuliana D’Auria, Carlo Cosimo Quattrocchi, Barnaba Floreno, Michele Fiore, Teresa Gamucci, Giuseppe Tonini, Sara Ramella

**Affiliations:** 1 Department of Radiation Oncology, Campus Bio-Medico University, 00128 Rome, Italy; s.silipigni@unicampus.it (S.S.); p.matteucci@unicampus.it (P.M.); c.greco@unicampus.it (C.G.); b.floreno@unicampus.it (B.F.); m.fiore@unicampus.it (M.F.); s.ramella@unicampus.it (S.R.); 2 Department of Medical Oncology, Campus Bio-Medico University, 00128 Rome, Italy; francesco.pantano@unicampus.it (F.P.); giuseppe.tonini@unicampus.it (G.T.); 3 Department of Medical Oncology, Sandro Pertini Hospital, 00157 Rome, Italy; g.dauria@aslroma2.it (G.D.); t.gamucci@aslroma2.it (T.G.); 4 Department of Diagnostic Imaging and Interventional Radiology, Campus Bio-Medico University, 00128 Rome, Italy; c.quattrocchi@unicampus.it

**Keywords:** SRT, breast cancer, dual blockade, trastuzumab, pertuzumab

## Abstract

**Simple Summary:**

Brain metastases (BM) may affect a large portion of metastatic HER2-positive BC patients. Dual human epidermal growth factor receptor 2 (HER2) blockade with Pertuzumab and Trastuzumab (PT) in conjunction with chemotherapy represents the first line therapy of metastatic HER2- positive breast cancer (BC) and shows to prolong time to BM development as the first site of disease progression. When limited brain disease occurs with stable extracranial disease, current guidelines suggest to continue systemic therapies and add local brain treatment. However not data are available on the association between PT and stereotactic brain radiotherapy (SRT). This study aims to assess the safety and efficacy of fractionated SRT (fSRT) and PT in patients with breast cancer BM.

**Abstract:**

(1) Background: This study aims to assess the safety and efficacy of fractionated SRT (fSRT) and pertuzumab–trastuzumab (PT) in patients with breast cancer brain metastases (BCBM). (2) Methods: Patients with HER2+ BCBM who received FSRT from 2015 to 2019 were identified. Patients were included if they were treated with fSRT within 21 days of receiving PT. All lesions were treated with LINAC-based fSRT to a total dose of 27 Gy delivered in three consecutive fractions. All patients received concurrent PT. Patients were evaluated 4–6 weeks after SRS and subsequently every 2–3 months with MRI re-imaging (3) Results: A total of 49 patients with HER2+ brain metastases were identified. Of these patients, a total of 10 patients with 32 HER2+ BCBM were treated with concurrent SRT and PT and included in the analysis. No local progression was observed. Overall response rate was 68.7%. Only one patient developed asymptomatic radionecrosis. Median time to BM occurrence was 15.6 (range: 1–40.5 months). Distant intracranial failure occurred in 4/10 patients (40.0%). Overall BCBM median survival was 33.9 months (95%CI 24.1–43.6). Mean duration of PT treatment was 27.9 months (range: 10.1–53.7 months). (4) Conclusions: In our single institution experience, fSRT and PT showed to be a safe treatment for patients with BCBM with an adequate overall response rate.

## 1. Introduction

In HER2-positive breast cancer (BC) metastatic patients, it is estimated that more than 30% of patients will develop brain metastases (BM). In the post-trastuzumab era, with patients experiencing longer survival due to increased extracranial disease control, the brain often represents the first site of relapse. Nevertheless, the introduction of HER2-targeted therapies improved the survival HER2 positive BC patients, including those with BM, ranging from 12 to 24 months [1,2,3,4,5].

Pertuzumab (P) is a humanized monoclonal antibody that is directed against extracellular domain 2 of HER2 receptor and inhibits the interaction of HER2 with HER3. Similarly to trastuzumab (T), P stimulates both antibody-dependent and cell-mediated cytotoxicity. In preclinical HER2 positive models, the T and P combination shows increased antitumor effect compared to either agent alone [6,7,8].

In the phase III CLEOPATRA study, the combination of P and T plus docetaxel showed a significant impact on progression-free survival and overall survival in HER2-positive metastatic BC patients [9]. The dual blockade with PT also showed to prolong time to BM development as the first site of disease progression and overall survival over trastuzumab alone in a post hoc, exploratory analysis from the CLEOPATRA study [10].

In the setting of BM, local treatment still represents the standard of care, particularly as radiotherapy has improved over the last years and stereotactic radiosurgery (SRS), as well as fractionated stereotactic radiotherapy (fSRT), have now become a non-invasive, effective alternative to surgical resection. Modern SRT also allows the treatment of patients with multiple BM with similar outcomes to those with 2–4 BM [11]. Also, carefully selected patients with 11–20 BMs may benefit from SRT [12]. The combination of SRS with systemic therapy permits potential synergistic benefit, but should be monitored for the risk of increased toxicity.

Preclinical studies showed that trastuzumab is an effective radiosensitizer [13,14] as breast cancer cells overexpressing HER2 are more radioresistant and blocking HER2 represents a way to sensitize cells to radiotherapy. Even if no such data is available for dual blockade, given that the dual blockade is more effective in overcoming resistance to a single blockade, it is also likely to be an even more powerful radiosensitizer. In addition, even if both T and P are large molecules with high molecular weight, studies using positron emission tomography-computed tomography (PET-CT) with Cu-DOTA-trastuzumab and 89Zr-pertuzumab showed some blood brain barrier penetration [15,16], leaving room for interaction.

Even if there are some data on the association of RT and PT in other settings such as palliative bone treatment and postoperative BC treatment [17,18,19] with a favourable tolerance profile, no literature data is available on the association between PT and brain fSRT.

For this reason, the aim of our study was to assess the safety and efficacy of the combination of trastuzumab, pertuzumab, and brain fSRT.

## 2. Materials and Methods

### 2.1. Study Design

This is a retrospective study aimed at assessing the safety and efficacy of fSRT and double blockade HER2 with pertuzumab and trastuzumab in patients with breast cancer brain metastases (BCBM), performed in accordance with the Declaration of Helsinki.

Patients with HER2+ BCBM who received fSRT from December 2015 to December 2019 were identified in the institutional database. Patients were included if they were treated with stereotactic radiation within 21 days of receiving PT (either before, during, or after administration). Based on institutional policy and local ethical committee requirements, patients already signed the informed consent for treatment and use of their clinical data for research or educational purposes.

### 2.2. Treatment

All lesions were treated with LINAC-based fSRT (TrueBeam STx linear accelerator, Varian Medical System, Palo Alto, CA, USA) using a commercial stereotactic mask fixation system (BrainLab AG, Munchen, Germany). Target volumes were contoured on 1 mm slice gadolinium-enhanced T1-weighted axial MRI sequences fused with planning computed tomography (CT) scans. The gross tumor volume (GTV) was delineated as the contrast-enhancing tumor demonstrated on MRI scans. The planning tumor volume (PTV) was generated, giving a geometric expansion to GTV of 0.5 mm. Planned total dose was 27 Gy delivered in three consecutive fractions [20]. Prescription dose was reduced in the case of lesions close to or within critical brain structures. Doses were prescribed to the 80% isodose line and delivered using noncoplanar volumetric arcs. Cone-beam CT and ExacTrac^®^ image-guided systems were used to ensure accurate patient positioning.

All patients received 6 mg/kg IV of concurrent trastuzumab once every 3 weeks plus pertuzumab administered IV 420 mg on the same day as trastuzumab.

### 2.3. Outcome Measures

Patients were evaluated during follow-up 4–6 weeks after fSRT and subsequently every 2–3 months with MRI re-imaging. Response was evaluated on a “per lesion” basis as in other studies investigating BM response to SRT [21,22,23]. For brain metastases measuring ≥ 5 mm, intracranial complete response (CR), partial response (PR), stable disease (SD), and progressive disease (PD) were determined by MRI according to the response evaluation criteria in solid tumors (RECIST v 1.1) as follows: CR was defined as the disappearance of the target lesion; PR has at least a 30% decrease in the sum of diameters of the target lesion; PD has at least a 20% increase in the sum of diameters of the target lesion; SD has neither sufficient shrinkage to qualify for PR nor sufficient increase to qualify for PD [24]. The overall objective response rate (ORR) was defined as the percentage of patients with intracranial CR and PR. Intracranial clinical benefit (iCB) was defined as percentage of patients with intracranial CR, PR, and SD. RN was defined as any enlarging lesion on T1 images with gadolinium enhancement, located at the place of a metastasis previously treated with SRS with a minimum delay of 6 months, with secondary characteristics not compatible with a local progression (i.e., T1–T2 mismatch; increase of the necrotic component or spontaneous regression on further MRI; hypoperfusion on dynamic susceptibility contrast (DSC) perfusion MRI).

Local failure was determined on a “per lesion” basis from the time of fSRT. Development of new BM based on follow-up imaging was defined on a “per patient basis” as distant intracranial failure. Overall survival was determined from the time of initial occurrence of BM. Follow-up period was calculated from the beginning of fSRT to the last observation.

### 2.4. Statistical Analysis

General descriptive statistical calculations were done on the current baseline and on treatment of clinical parameters. Continuous variables were summarized with median and range, whereas counts and percentages were used to summarize categorical variables. The Kaplan–Meier (KM) method was used to estimate survival outcomes. All statistical analyses were performed using IBM SPSS 23 software (IBM, SPSS Statistics, Chicago, IL, USA).

## 3. Results

### 3.1. Study Population

A total of 49 patients with HER2+ brain metastases were identified. The majority of these patients were treated with fSRT given concurrently with Trastuzumab (59.2%) and to a lesser extent with fSRT in association with Lapatinib (20.4%). Of these patients, a total of 10 patients (20.4%) with 32 HER2+ BCBM were treated with concurrent fSRT and PT over 17 treatment sessions and were included in this analysis. The median number of lesions treated in each patient was two (range 1–9). None of these patients received any additional concurrent chemotherapy. The majority of patients were HR+/HER2+ (6/10; 60.0%). Patients’ characteristics are summarized in Table 1.

The median dose of FSRT was 27 Gy (range 12–27 Gy) with a median dose per fraction of 9 Gy (range 4–9 Gy). For nine lesions, the prescription dose was lowered due to proximity to critical organs (brainstem in five cases, optic nerves in three cases, and optic chiasma in one case). The median PTV of lesions was 2.26 cm^3^ (range: 0.8–54.4). Brain metastases were mainly located in the brain region supplied by the posterior circulation (20/32; 62.5%). See Table 2 for details.

### 3.2. Efficacy

With a median follow-up of 18.3 months (range: 1.5–39.8 months), no local progression in lesions treated was observed. Overall response rate was 68.7%. We also recorded 10 stable lesions (31.3%). The The iCB observed was 100%.

### 3.3. Toxicity

**Figure 1:**
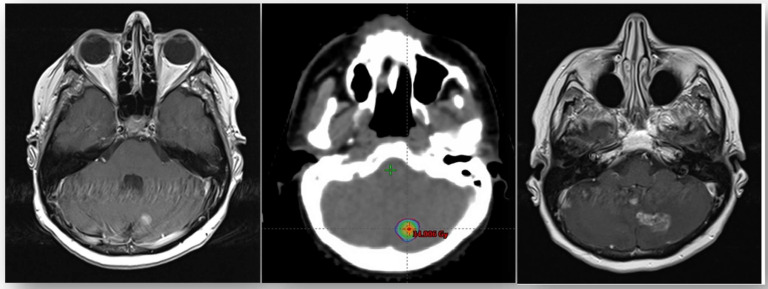
Brain MRI showing cerebral radionecrosis: (Left): T1-sequence MRI showing left cerebellar metastasis; (Middle) fSRT treatment planning; (Right) 12 months T1-sequence MRI: radionecrosis appearance. No other toxicity was experienced during treatment. No patient suspended PT.

Only one patient developed radionecrosis to one cerebellar lesion. The lesion was >1 cm, and the patient developed clinical asymptomatic radionecrosis 12 months after fSRT (Figure 1).

### 3.4. Outcome

**Figure 2:**
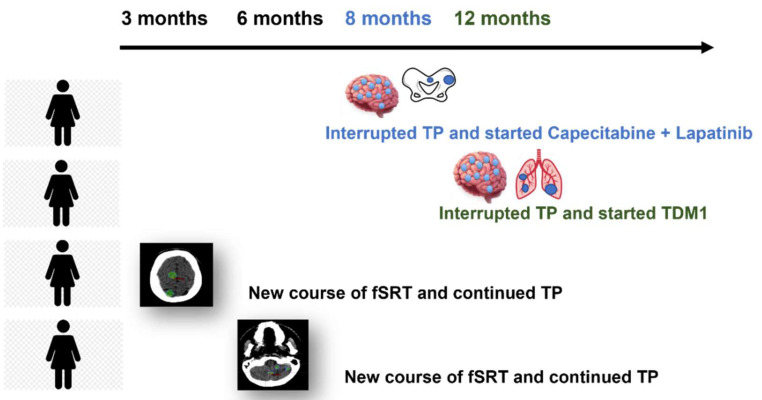
Graphical representation of distant intracranial pattern of failure observed in four patients (see text for details).

Median follow-up was 18.3 months (range: 1.5–39.8 months). Median time to BM occurrence from the beginning of PT was 15.6 (range: 1–40.5 months). Distant intracranial failure after fSRT occurred in 4/10 patients (40.0%). In two patients, multiple brain lesions (>20) were observed after 1 year and after 8 months, respectively, concurrently with extracranial disease progression; for this reason, systemic treatment was changed (one to lapatinib–capecitabine, one to trastuzumab–emtansine); in one patient, two new lesions were observed at the first follow-up MRI after fSRT, leading to new courses of fSRT; in another patient, three new lesions appeared after 6 months and were treated with a subsequent course of fSRT (see Figure 2).

Overall brain metastases median survival was 33.9 months (95%CI 24.1–43.6). Mean duration of PT treatment in these patients was 27.9 months (range: 10.1–53.7 months).

## 4. Discussion

The primary goal of our study was to assess the efficacy and tolerance of the combination of trastuzumab, pertuzumab, and brain fSRT. The treatment combination in our single institution population showed to be safe and effective. Very few data are available on the association between PT and brain radiotherapy, and none on the association with fSRT.

In the phase III CLEOPATRA study, 13.7% of patients (55/402) of the PT arm developed CNS metastases, with a median time to brain progression of 15.0 months vs. 11.9 months in the placebo arm (HR = 0.58, 95% CI 0.39–0.85, *P* = 0.0049). Of these, 23 patients received radiotherapy to the brain and only 5 gammaknife SRS [10]. In a more recent analysis of 252 HER2-positive BC patients, only 26 patients were treated with dual blockade and 14 patients were evaluable for response. Of these, 12 patients underwent local therapy (either surgery or WBRT or SRS). The ORR and iCBR were 92.9% and 100.0% respectively. The only patient who did not receive local therapy (surgery, WBRT or SRT) reported a PR [25].

Current guidelines suggest that for those patients whose systemic disease is not progressive at the time of brain metastasis diagnosis, they should not switch systemic therapy and refer the patients for radiotherapy [26]. However, as patients with BM are often excluded from registration trials and little data is available regarding local treatments, limited knowledge is available on the interaction between brain radiotherapy and novel drugs. Indeed, retrospective analyses may be of interest.

Ajgel et al., conducted a retrospective study on the safety of radiotherapy associated with dual blockade in a population of 23 consecutive BC patients between 2013 and 2015. Radiotherapy was performed on the chest area or metastatic sites (one patient underwent whole brain radiotherapy). Treatments were well tolerated with one patient experiencing a decrease in left ventricular ejection rate and only one patient showing grade 3 skin toxicity [18]. Dreyfuss et al. reported radiation-related toxicities in 80 patients receiving hypofractionated whole breast radiation with concurrent PT. There was one grade 3 acute toxicity (fatigue) and no grade 3 late toxicities [17]. More recently, in a large retrospective cohort of 55 patients, the combination of locoregional breast RT with PT resulted as well tolerated. Particularly, only three cases of grade 3 skin toxicity were reported, suggesting a good safety profile for the association [27].

In the largest retrospective series reporting the outcome of HER2 BC patients treated with brain stereotactic treatment, no patient was treated with Pertuzumab. An ORR of 72% was reported for patients receiving fSRT alone [23]. In this series, the role of lapatinib in association with radiation treatment was mainly investigated. Lapatinib given concurrently with brain stereotactic radiotherapy showed not only a decreased local failure rate (5.7% vs. 15.1%, *p* < 0.01) but was also associated with a reduced rate of RN (1.3% vs. 6.3%, *p* < 0.01) compared to SRT alone [28,29].

In our series, the overall response rate was 68.7% with a similar efficacy of fSRT alone.

Only one patient developed asymptomatic RN to one large cerebellar lesion. No other neurological symptoms were recorded, nor other toxicity. As the occurrence of RN is multifactorial and increases linearly with lesion size, it is likely that the size of lesion has primarily influenced this toxicity [30,31]. Moreover, the overall brain metastases-free survival and the duration of PT treatment are consistent with literature data and support the use of brain local treatment to prolong the extracranial benefit of dual blockade [9,10].

Interestingly, in our series, some patients were treated to multiple intracranial sites (median lesions per patients: two, range: one to nine) also at the same time. Therefore, the fSRT can be a valid option also for patients with multiple cerebral sites of disease for whom sometimes a switch in systemic therapy is preferred in order to avoid whole brain radiotherapy and its late neurocognitive effects.

This study has several limitations such as its retrospective nature, small sample size, and short follow-up. In fact, longer follow-up is needed especially to assess RN rate, which usually develops from 6 months to several years after fSRT [31]. Moreover, even if the combination seems effective, this analysis does not allow for a direct comparison with other associated anti HER2 drugs or with PT alone. Therefore, while clinically interesting and novel, our results need to be interpreted with caution.

## 5. Conclusions

In our single institution experience, fSRT and PT showed to be a safe treatment for patients with BCBM with an adequate overall response rate. Overall brain metastases-free survival and the duration of PT treatment appear to be similar to literature data and support the use of brain local treatment to prolong the extracranial benefit of dual blockade.

## Figures and Tables

**Table 1:** Patients’ Characteristics.

Variable	n	%
Total patients	10	100
Age		
Median	57	
Range	42–78	
Receptors		
HR+/HER2+	6	60.0
HR−/HER2+	4	40.0
Total FSRT courses	17	100
Lesions treated per patient		
Median	2	
Range	1–9	-
Breast GPA group		
0.0–1.0	0	
1.5–2.0	2	20.0
2.5–3.0	7	70.0
3.5–4.0	1	10.0

FSRT = fractionated stereotactic radiotherapy; GPA = graded prognostic assessment.

**Table 2:** Treatment data.

Variable	n	%
Brain fSRT	32	100
PTV		
Median	2.26 cm^3^	
Range	0.8–54.4	
Brain Metastases Location		
Parietal lobes	5/32	15.6
Frontal lobes	7/32	21.9
Occipital lobes	2/32	6.3
Temporal lobes	8/32	25.0
Cerebellum	5/32	15.6
Brainstem	5/32	15.6
Total dose		
Median	27	-
Range	12–27	
Dose per fraction		
Median	9	
Range	4–9	
Response (RECIST criteria)		
CR	14/32	43.7
PR	8/32	25.0
SD	10/32	31.3
PD	0/32	0.0

## Data Availability

Research data are stored in an institutional repository and will be shared upon request to the corresponding author.
